# Integrating physiological indicators and Precision Livestock Farming to support European animal welfare frameworks: a systematic review

**DOI:** 10.1080/01652176.2026.2739038

**Published:** 2026-11-04

**Authors:** Giulia Pastorelli, Rita Iacono, Alda Quattrone, Giacomo Contò

**Affiliations:** a CREA Research Centre for Agricultural Policies and Bioeconomy, Rome, Italy; b Department of Veterinary Medicine and Animal Sciences, University of Milan, Lodi, Italy; c CREA Research Centre for Animal Production and Aquaculture, Rome, Italy

**Keywords:** Animal welfare, physiological measurements, key indicators, Precision Livestock Farming, non-invasive monitoring, biosensors, animal welfare monitoring, European animal welfare framework

## Abstract

Animal welfare assessment in livestock production is increasingly supported by Precision Livestock Farming (PLF) technologies integrated with physiological monitoring. This Scopus-based systematic literature review, conducted according to PRISMA guidelines and synthesised using a narrative approach, examined physiological parameters relevant to animal welfare assessment and their integration into PLF across major livestock species, including cattle, pigs, poultry, sheep, and goats. Following the screening and eligibility assessment, 144 studies were selected from an initial dataset of 1386 records. Body surface temperature, heart rate, and respiratory rate were the most frequently investigated physiological parameters, while computer vision, wearable sensors, environmental sensing, and machine learning were the predominant technologies enabling continuous, non-invasive monitoring. The reviewed studies primarily addressed animal-based welfare indicators within European welfare assessment frameworks, whereas resource- and management-based indicators were comparatively underrepresented. No studies explicitly investigated antimicrobial use or the human–animal relationship using PLF technologies, highlighting important research gaps. Overall, the evidence indicates that physiological parameters provide complementary biological information that enhances the interpretation of animal-based welfare indicators, particularly when integrated with behavioural and environmental monitoring. Future research should prioritise multimodal approaches integrating physiological, behavioural, environmental, and management-related data to support more comprehensive and operationally feasible animal welfare assessment.

## 1. Introduction

Animal welfare is a multidimensional concept encompassing not only the absence of suffering and disease but also the maintenance of adequate biological functioning and the provision of opportunities for animals to experience positive physical and emotional states (Fraser 1993). It reflects both the physical and mental condition of the animal, as well as its ability to express natural behaviours and cope with environmental challenges (Hewson 2003).

Over recent decades, animal welfare has become a central component of European Union (EU) agricultural and animal health policies, driven by its ethical, societal, and public health implications. Increasing attention has also been paid to the interconnections between animal welfare, food safety, product quality, and the sustainability of livestock production systems. Within this framework, the European Green Deal places animal welfare at the core of sustainable food systems. Improving animal welfare is considered a key strategy to enhance animal health and reduce the reliance on antimicrobial use in livestock production, thereby contributing to the mitigation of antimicrobial resistance and promoting more sustainable farming practices (European Commission 2018).

Despite this strong policy framework, the definition and implementation of harmonised animal welfare standards across the EU remains challenging due to the marked heterogeneity of farming systems, climatic conditions, and territorial characteristics among Member States. These differences influence both the applicability of legislative measures and the feasibility of standardised welfare assessment protocols, highlighting the need for scientifically robust, flexible, and practical assessment approaches. In this context, animal welfare assessment methodologies have evolved significantly over time. The traditional ‘Five Freedoms’ model, originally developed by the UK Farm Animal Welfare Council in the 1960s and 1970s, provided a foundational approach focused on the prevention of negative states, including hunger, discomfort, pain, fear, and restrictions on the expression of natural behaviours (Farm Animal Welfare Council (FAWC) 2009). While highly influential in shaping both legislation and public perception, this concept has been progressively complemented by more comprehensive models that better capture the complexity of animal welfare. Among these, the ‘Five Domains’ framework (Mellor et al. 2020) provides a more complete evaluation of animal welfare, considering both physical and affective states across five dimensions: nutrition, environment, health, behaviour, and mental state.

To translate these concepts into practical animal welfare assessment tools, the European Welfare Quality project developed standardised, animal-based protocols for major livestock species, including cattle, pigs, and poultry (Dai and Battini, 2016). These protocols are structured around four core principles: good nutrition, good housing, good health, and appropriate behaviour, which are further subdivided into twelve criteria assessed through direct observation of animal health and behaviour. Following this methodology, the AWIN (Animal Welfare Indicators) project subsequently extended this approach to other commercially important species, including small ruminants, by adapting the assessment protocols to species-specific behavioural and physiological characteristics.

Building upon these foundational tools, the ‘EU Strategy for the Protection and Welfare of Animals 2012–2015’ (European Commission 2012) represented a pivotal step toward promoting an outcome-oriented approach aimed at improving livestock conditions through regulatory measures and effective assessment systems. Subsequently, the European Commission evaluated the impact of Common Agricultural Policy (CAP) measures on animal welfare and antimicrobial use across the principal livestock sectors during the 2014–2020 programming period (European Commission 2022). This work culminated in the publication of the ‘Framework for Animal Welfare Indicators under the CAP 2023–2027’ (European Commission 2024), which established a harmonised set of 13 key indicators to support the evaluation of animal welfare practices and policy implementation across EU Member States. These 13 indicators encompass animal-, resource-, and management-based measures. Animal-based indicators directly evaluate the animals' health, physical condition, and behavioural responses. They include health-related measures such as the absence of injuries, lameness, body condition score, metabolic health, mortality, and integument condition, together with behavioural measures including locomotion, resting comfort, social behaviour, and the human–animal relationship. Resource-based indicators assess the physical and environmental characteristics of the animals' surroundings that may influence their welfare, including thermal conditions, stocking density, housing design, flooring, air quality, environmental enrichment, and access to feed and water. Management-based indicators evaluate husbandry practices and farm management decisions, including housing systems and antimicrobial use.

The relevance of the 2024 European framework extends beyond a regulatory update, as it provides one of the first harmonised and policy-oriented structures for integrating animal-, resource-, and management-based indicators across livestock systems within the European Union. These complementary categories capture different dimensions of animal welfare: animal-based indicators evaluate the animals' health, physical condition, and behavioural responses, whereas resource- and management-based indicators assess the environmental conditions and husbandry practices that influence these outcomes. As such, the framework provides a valuable reference for evaluating how emerging physiological measurements and Precision Livestock Farming (PLF) technologies can complement existing welfare assessment approaches.

Crucially, animal welfare monitoring is also closely linked to EU policies aimed at reducing antimicrobial use. Within the CAP 2023-2027 programming period, improving animal welfare and reducing reliance on antimicrobials remain key priorities, reflecting the broader One Health approach to tackling antimicrobial resistance (WHO 2017). In this regard, enhanced cross-compliance has been integrated into the CAP, establishing a binding framework of environmental, climate, and safety standards. This framework encompasses Statutory Management Requirements (SMRs) and Good Agricultural and Environmental Conditions (GAECs), which farmers must comply with to access direct payments and support under Rural Development Programmes (RDPs). Notably, the system also incorporates specific animal welfare provisions, including strict rules on the protection of calves (Directive 2008/119/EC), pigs (Directive 2008/120/EC), and animals kept for farming purposes more broadly (Directive 98/58/EC).

Despite these important policy developments, selecting appropriate welfare indicators remains a major challenge. Recent work has emphasised that effective welfare assessment should rely on carefully optimised combinations of complementary indicators that balance biological relevance, coverage of different welfare dimensions, operational feasibility, and practical implementation, rather than simply increasing the number of individual measures (Day et al. 2025). Contemporary assessment frameworks primarily evaluate welfare using animal-, resource-, and management-based indicators. Although these provide valuable information on welfare outcomes and the conditions influencing them, they may not fully capture the animals' biological responses to environmental and management challenges. Physiological measurements reflecting thermoregulation, cardiovascular function, respiratory activity, endocrine responses, and metabolic status, represent complementary animal-based measurements that can enhance the biological interpretation of behavioural and clinical observations. When integrated with behavioural and environmental information, they may also provide early indications of compromised welfare before overt clinical signs become apparent.

In this context, Precision Livestock Farming (PLF) has emerged as an innovative and rapidly evolving approach for the continuous, automated, and individual-based monitoring and management of livestock. Rather than representing merely a collection of sensing technologies, PLF functions as a decision-support framework that integrates sensors, artificial intelligence, advanced analytics, and digital tools to generate real-time information for informed management decisions (Berckmans 2017; Kleen and Guatteo 2023). By combining wearable biosensors, computer vision, infra-red thermography, acoustic monitoring, environmental sensing, and production records, PLF enables the non-invasive acquisition of complementary physiological, behavioural, environmental, and production-related information. This multidisciplinary approach supports the transition from reactive herd management to proactive, evidence-based management, improving the capacity to monitor animal welfare while contributing to more informed livestock management (Schillings et al. 2023).

Current PLF applications predominantly support the automated monitoring of animal-based welfare indicators, particularly behavioural measures such as locomotion, feeding behaviour, rumination, resting behaviour, and social interactions, which can be continuously monitored using computer vision, acoustic analysis, and wearable sensing technologies. In contrast, physiological measurements, including heart rate (HR), heart rate variability (HRV), respiratory rate (RR), body surface temperature, glucocorticoids, and other biomarkers, provide complementary information on the biological responses underlying these observable welfare outcomes. Rather than constituting independent welfare indicators, physiological measurements enhance the interpretation of behavioural and health-related observations by providing objective information on the biological processes underlying observable welfare outcomes. Their integration with behavioural, environmental, and production-related data therefore has the potential to support earlier detection and more biologically informed assessment of compromised welfare (Kleen and Guatteo 2023). However, the routine implementation of physiological monitoring within PLF depends not only on the biological relevance of individual measurements but also on their technological feasibility, robustness, and scalability under commercial farming conditions. Consequently, successful implementation requires close collaboration among veterinarians, animal physiologists, engineers, and data scientists (Berckmans 2017).

Although PLF research has expanded considerably in recent years, existing reviews have largely focused on technological innovations, sensing platforms, artificial intelligence, and broader research trends. For example, the global scientometric analysis by Jiang et al. (2023) identified deep learning, computer vision, intelligent systems, and behavioural monitoring among the major research hotspots, highlighting the increasingly multidisciplinary nature of the field. Conversely, reviews dedicated to physiological parameters have generally examined these measures independently of their practical application in PLF.

To address these knowledge gaps, we conducted a Scopus-based systematic literature review following PRISMA guidelines and synthesised the evidence using a narrative approach. The literature search covered publications from 2015 to 2025 across major livestock species and applied predefined inclusion and exclusion criteria. The primary aim of this review was to systematically identify and critically synthesise non-invasive physiological parameters relevant to livestock welfare assessment and examine their practical integration into Precision Livestock Farming technologies. In addition, the review evaluates how PLF applications address the animal-, resource-, and management-based indicators included in the European Commission Framework for Animal Welfare Indicators, thereby providing a comprehensive overview of the current alignment between technological developments and contemporary European welfare assessment frameworks. By combining veterinary, engineering, and policy perspectives, this study seeks to identify current research gaps and highlight future opportunities for developing biologically meaningful, operationally feasible, and practically applicable welfare monitoring systems.

## 2. Methodology

The present study was designed as a Scopus-based systematic literature review aimed at identifying the main non-invasive physiological measurements used for livestock welfare assessment and evaluating the measurement approaches and their integration into Precision Livestock Farming (PLF) technologies. The review was conducted according to the methodological principles outlined in the Cochrane Handbook for Systematic Reviews of Interventions (Cumpston et al. 2024), ensuring transparency, reproducibility, and methodological rigour throughout the selection process. The review protocol was not formally registered prior to conducting the study.

The PICO framework (Population, Intervention, Comparison, Outcome) was adopted (Table 1) to define the scope of the review and guide the development of the search strategy (Sala et al. 2006). The population considered included livestock production systems, specifically bovine, swine, ovine, caprine, buffalo, and poultry species. The intervention focused on non-invasive physiological parameters and the measurement approaches used to assess or validate animal welfare status, with particular emphasis on techniques compatible with PLF systems. The comparison addressed differences between invasive and non-invasive physiological measurements applied to animal welfare assessment. The outcome was the identification of validated physiological measurements associated with animal welfare and their potential integration into PLF-based monitoring systems.

**Table 1.** PICO framework used to define the review scope.

P	Population/Problem	Livestock production systems
I	Intervention	Physiological parameters and the corresponding measurement methods used to assess or validate animal welfare status, with particular emphasis on non-invasive, scalable techniques and approaches potentially compatible with Precision Livestock Farming (PLF) systems.
C	Comparison	Comparison between invasive and non-invasive physiological measurements applied to animal welfare assessment in livestock systems
O	Outcome	Scientific evidence identifying the non-invasive physiological measurements most strongly associated with animal welfare, and their potential integration into PLF-based monitoring frameworks

Source: Author’s elaboration.

To ensure methodological rigour, inclusion and exclusion criteria (Table 2) were defined a priori according to established guidelines for systematic reviews in veterinary and animal sciences (Sargeant et al. 2005). Studies were eligible if they evaluated non-invasive physiological measurements related to animal welfare in livestock species, were published in English as original research or review articles between 2015 and 2025 and were available in full text. Conversely, studies were excluded if they focused exclusively on invasive measurements, involved non-livestock species, or did not directly address animal welfare indicators.

**Table 2.** Eligibility criteria adopted for study selection.

Domain	Inclusion criteria	Exclusion criteria
Topic	Studies evaluating non-invasive physiological measurements related to animal welfare in livestock systems, including parameters objectively associated with EU-identified welfare indicators	Studies not addressing animal welfare or focusing exclusively on invasive measurements
Population (species)	Studies conducted on major livestock species: bovine (dairy and beef), swine, ovine, buffalo, caprine, broiler chickens and laying hens	Studies conducted on companion animals, equines (including horses), fish, wildlife or other non-livestock species
Study type	Primary research articles and peer-reviewed reviews	Editorials, commentaries, opinion papers, and conference abstracts
Language	English	All other languages
Timeframe	Published between 2015 and 2025	Publications prior to 2015
Availability	Full-text available (via open access or institutional subscription)	Abstract-only records or inaccessible full texts

Source: Author’s elaboration.

The literature search was conducted using the Scopus database and included publications indexed up to June 2025. Although the use of multiple databases is generally recommended to maximise coverage, Scopus was considered appropriate for this systematic review due to its broad multidisciplinary scope, encompassing agricultural, veterinary, and animal sciences, its large volume of indexed records, and its rigorous content selection process based on transparent criteria. These characteristics ensure a high level of consistency and reliability in the identification and classification of the selected literature. Furthermore, the use of a single database facilitated methodological consistency across the two integrated search strategies adopted in the review. Two complementary search strings were applied: (‘animal welfare’ AND ‘physiological measurements’) and (‘animal welfare’ AND ‘precision livestock farming’). Both search strings were applied within a single, integrated systematic review framework and subjected to the same inclusion and exclusion criteria. This dual-search strategy was designed to capture complementary dimensions of the research topic, namely the physiological validation of animal welfare indicators and their application within PLF systems. Specifically, the first search string focused on identifying physiological parameters associated with animal welfare and the methods used to measure them, whereas the second focused on the application of PLF technologies for monitoring animal-, resource-, and management-based welfare indicators. Although these two dimensions are closely interconnected, they were analytically distinguished throughout the review to separately evaluate physiological evidence and technology-based applications.

The study selection process followed the PRISMA (Preferred Reporting Items for Systematic Reviews and Meta-Analyses) guidelines (Page et al. 2021) and is illustrated in Figure 1.

Preliminary exclusion criteria (publication period, document type, language, and full-text availability) were applied directly through Scopus database filters. After this initial screening step, 700 records were retained for further evaluation.

**Figure 1.**
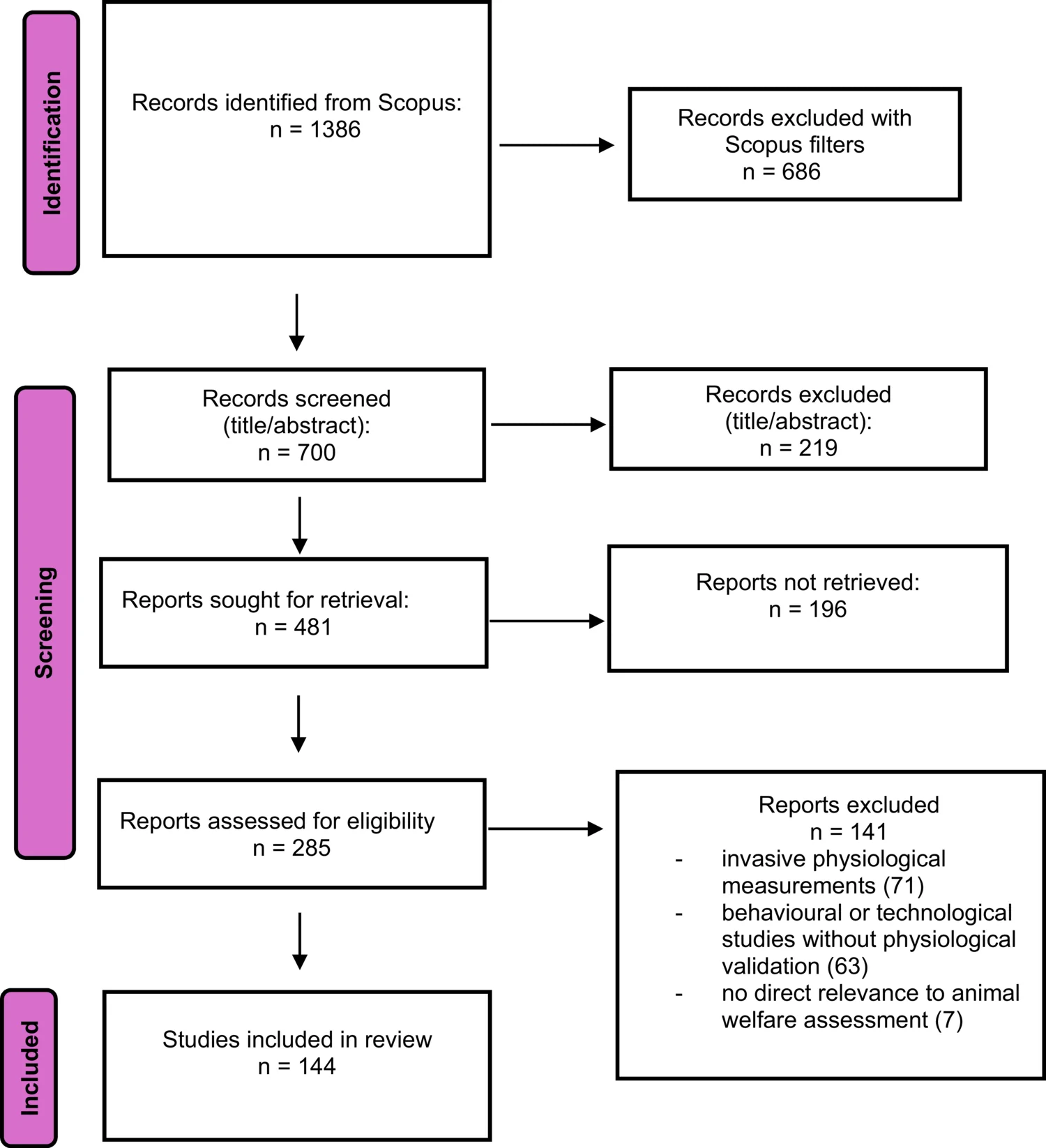
PRISMA flow diagram illustrating the study selection process. Source: author’s elaboration adapted from the PRISMA 2020 statement (Page et al. 2021).

Records were initially screened based on title and abstract, resulting in the exclusion of 219 studies that were outside the scope of the review, did not involve livestock species, or did not address relevant animal welfare indicators.

A total of 481 reports were then sought for retrieval, of which 196 were not retained at this stage due to lack of full-text availability (non-open access) or insufficient relevance to the research scope.

The remaining 285 articles were assessed for eligibility through full-text evaluation. During this phase, 141 reports were excluded due to the absence of non-invasive physiological measurements, a focus on behavioural or technological aspects without physiological validation, the use of invasive methods, or lack of direct relevance to animal welfare indicators.

Following the complete selection process, a total of 144 studies were included in the final review. Because several studies addressed multiple physiological parameters or welfare indicators, individual articles were categorised into more than one analytical category. Prior to synthesis, the selected studies were systematically classified into structured tables according to key analytical dimensions. A meta-analysis was not performed due to the high heterogeneity of livestock species, physiological parameters, sensor technologies, study designs, and welfare assessment approaches included in the selected literature. Consequently, the present review represents a first exploratory step toward systematically integrating physiological parameters with behavioural, environmental, and management-related information within PLF-based animal welfare assessment frameworks. Given the variability of study designs and measurement approaches, the results were synthesised using a narrative approach, ensuring a comprehensive and structured synthesis of both physiological and technology-based evidence on animal welfare assessment. A formal risk-of-bias assessment was not conducted due to the high heterogeneity of study designs, livestock species, physiological parameters, and PLF technologies included in the review, which limited the applicability of a single standardised assessment tool across all selected studies.

## 3. Results

### 3.1 Temporal and geographical distribution of literature by search string

As part of the descriptive analysis, the temporal evolution of the scientific literature was examined to identify trends in publication output over time for the two search strategies, considering the full set of records retrieved without temporal restrictions.

Figure 2 illustrates the annual distribution of publications retrieved through the two search strings. Distinct differences emerged in both the temporal development and growth patterns of the two research streams. Studies combining animal welfare and physiological measurements have been reported since 1980 and showed a relatively gradual and continuous increase over time. In contrast, publications investigating animal welfare in relation to PLF emerged only after 2001, with limited scientific output until the early 2010s, followed by a marked and sustained increase from 2017 onwards. In recent years, the number of PLF-related publications has progressively exceeded those focused exclusively on physiological measurements, reaching its highest levels during the 2024–2025 period.

**Figure 2.**
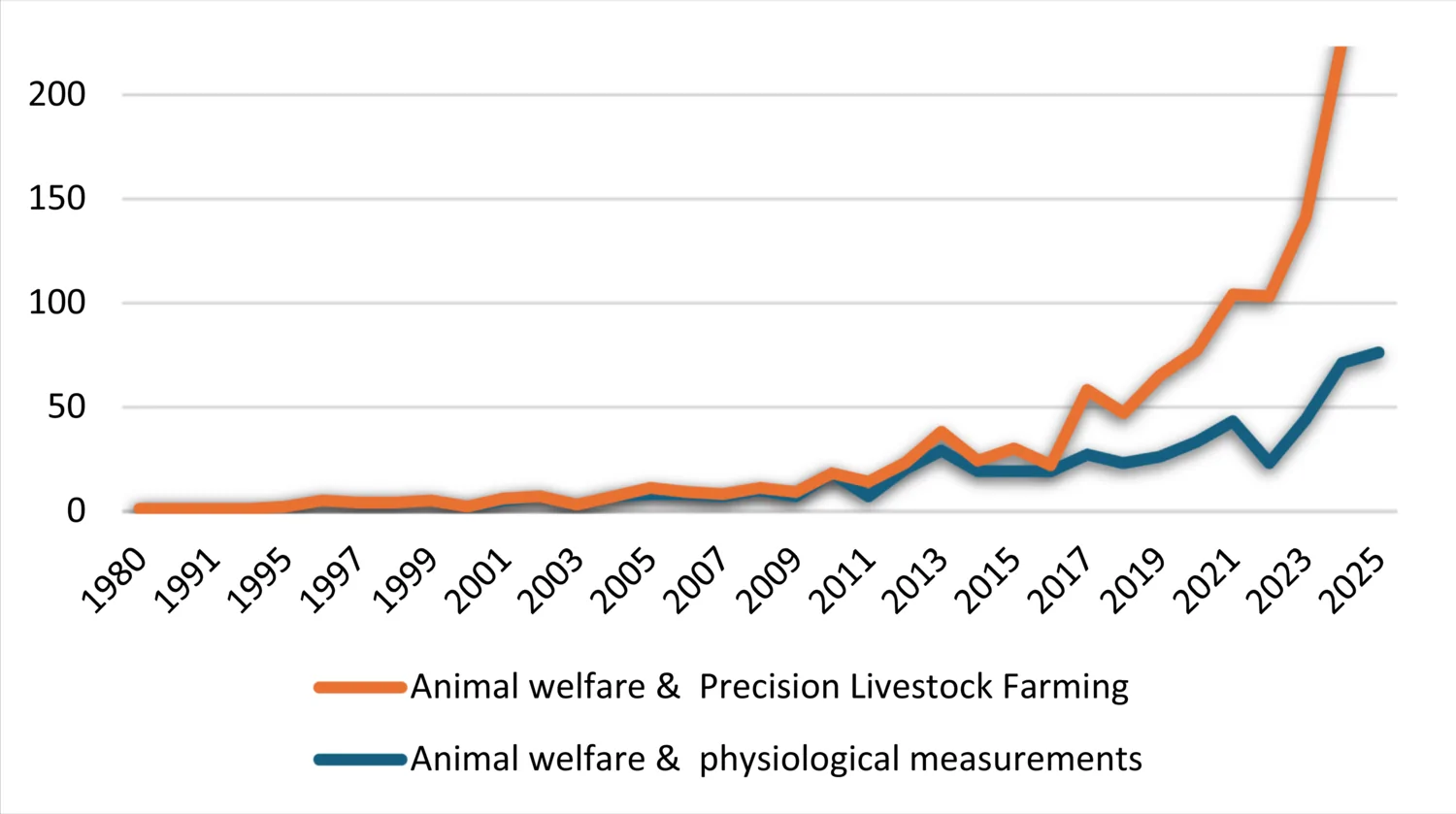
Annual distribution of publications by search string. Source: Author’s elaboration based on Scopus data.

Overall, both research streams showed a strong concentration of publications during the last decade. However, while studies investigating physiological parameters relevant to animal welfare demonstrated a gradual and continuous increase over time, PLF-related research exhibited a markedly faster growth trajectory, particularly after 2020. This pattern is consistent with the increasing application of sensor-based monitoring systems, automated data collection, and advanced data analytics in animal welfare research.

To further characterise the temporal distribution of the literature, a second analytical step focused specifically on studies published in the most recent decade, applying a temporal filter (2015–2025). Table 3 summarises the distribution of articles by search string before and after the application of the time filter.

**Table 3.** Temporal distribution of retrieved publications before and after application of the temporal filter (2015–2025).

Search string	No. of articles (no filters)	% of total	Articles with filters applied (2015–2025)	% of category
Animal welfare AND physiological measurements	585	42%	260	37%
Animal welfare AND Precision Livestock Farming	801	58%	440	63%
**Total**	**1.386**	**100%**	**700**	–

Source: Author’s elaboration based on Scopus data.

For the search string ‘*Animal welfare AND physiological measurements*’, the results indicated that a substantial proportion of the scientific production on physiological parameters used for animal welfare assessment has been published during the last ten years, reflecting the increasing relevance of objective and measurable approaches to welfare assessment in livestock systems. An even stronger temporal concentration was observed for the search string *“Animal welfare AND Precision Livestock Farming’*, with most publications falling within the 2015–2025 period. This finding suggests that the integration of digital technologies, sensor-based systems, and automated monitoring tools into animal welfare research is a relatively recent and rapidly expanding field.

To further characterise the literature retrieved using the search string ‘*Animal welfare AND Precision Livestock Farming*’, the geographical distribution of publications was analysed based on authors’ affiliations. As shown in Figure 3, research output was strongly concentrated within a limited number of countries.

**Figure 3.**
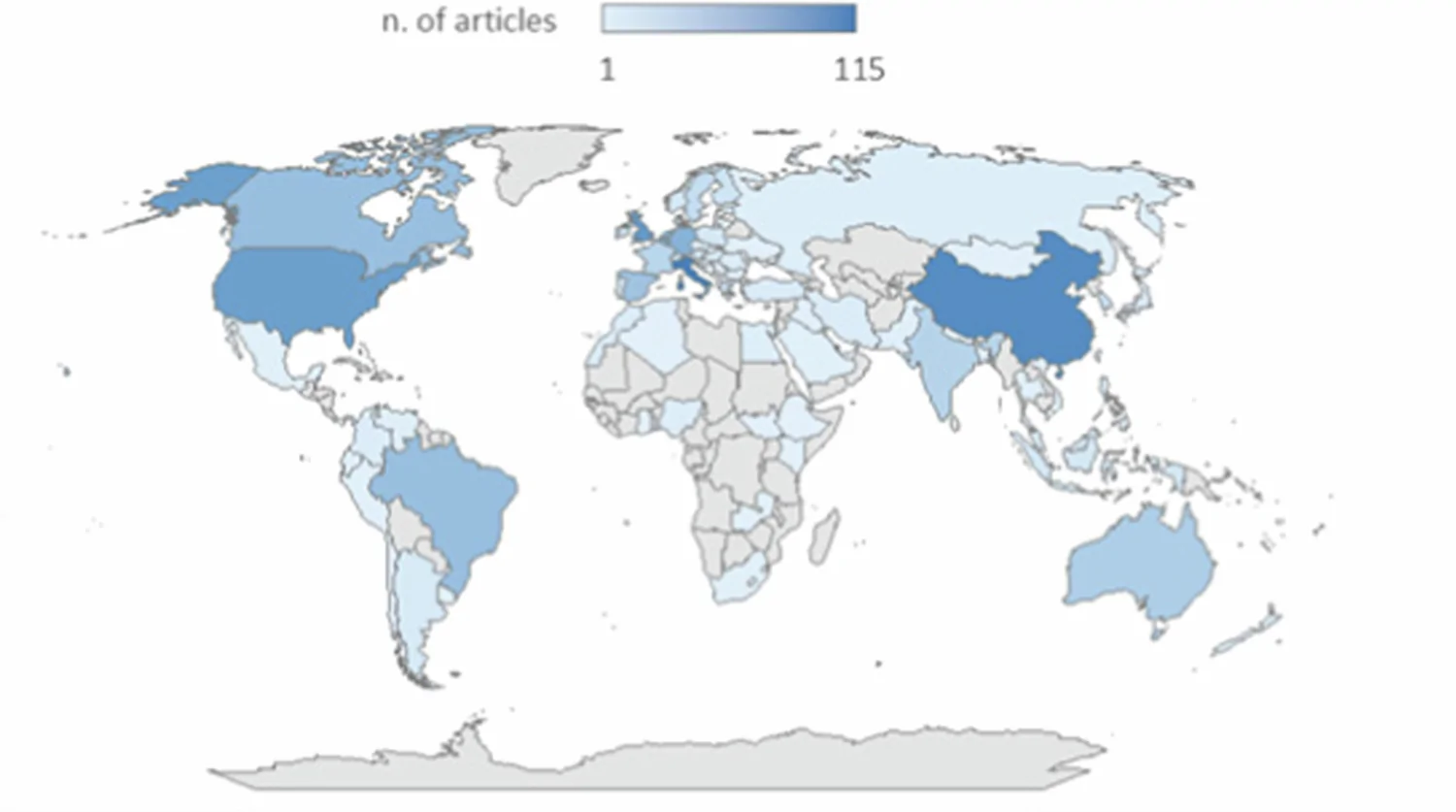
Geographical distribution of PLF-related animal welfare literature. Source: Author’s elaboration based on Scopus data.

Italy emerged as the leading contributor (115 publications), followed by China (102), the United Kingdom (87), and the United States (81). A strong European presence was also evident, with substantial contributions from Belgium (71), Germany (62), and the Netherlands (58), highlighting Europe as a major hub for research on PLF applications in animal welfare.

Outside of Europe and North America, notable scientific production was identified in Brazil and Canada (50 publications each), Australia (36), and India (30), suggesting growing global interest in PLF-based approaches for animal welfare monitoring, although with considerable regional variability. It should be noted that the geographical distribution was based on authors’ institutional affiliations; consequently, individual publications may have contributed to the publication output of multiple countries.

### 3.2 Identification and narrative description of physiological parameters in livestock welfare assessment

The screening of studies retrieved through the search string ‘*Animal Welfare AND Physiological Measurements*’ enabled the identification of several non-invasive physiological parameters currently applied in the assessment of animal welfare in livestock systems (Table 4). The reported frequencies refer exclusively to the studies included after the screening process and therefore represent the distribution of physiological parameters within the analysed sample rather than the entire body of literature. As some studies evaluated more than one physiological parameter, individual articles may contribute to multiple categories.

**Table 4.** Frequency distribution of non-invasive physiological parameters applied in livestock welfare assessment across the retrieved literature (2015–2025).

Physiological parameter	No. of studies
Body surface temperature (infra-red thermography - IRT)	24
Heart rate (HR)	21
Respiratory rate (RR)	20
Glucocorticoids (cortisol/corticosterone; saliva, hair, faeces)	14
Heart rate variability (HRV)	4
Oxytocin (saliva)	1
Calprotectin (saliva)	1

Source: Author’s elaboration.

Among the identified physiological parameters, body surface temperature measured through infra-red thermography (IRT) was the most frequently reported physiological parameter (24 studies). Similar findings were reported by Navia et al. (2024), who identified body surface temperature as the most commonly monitored physiological parameter, followed by heart rate (HR), while also highlighting the need for further development of devices capable of monitoring additional physiological variables, including blood pressure and blood oxygen saturation. Several studies applied IRT to measure body surface temperature as a non-invasive physiological parameter associated with physiological stress, health status, and thermal comfort across livestock species. Applications included dairy cattle, calves, and poultry, with particular attention to thermal stress assessment, the identification of highly vascularised anatomical regions, and the optimisation of imaging conditions for accurate temperature monitoring (Mostaço et al., 2015; de Sousa et al. 2016; de Oliveira et al. 2018; Coloma-García et al. 2020; Hernández-Sánchez et al. 2024; Song et al. 2025; Kim et al. 2025; Mitchaothai et al. 2025). Additional review studies further confirmed the relevance of body temperature monitoring for livestock welfare assessment under heat stress conditions (Polsky and von Keyserlingk 2017; Galán et al. 2018; Colditz 2022). The widespread use of IRT reflects its major advantage of capturing immediate, contactless thermal maps of animals from a safe distance, effectively reducing the need for animal restraint during measurement and enabling scalable monitoring. However, it only measures surface temperature, which can differ significantly from core body temperature, and its accuracy remains highly vulnerable to environmental factors (such as direct sunlight and wind speed), coat characteristics, anatomical region selection, and camera calibration, requiring careful interpretation.

Heart rate (HR) was the second most frequently reported physiological parameter, appearing in 21 studies. As an indicator of cardiovascular responses mediated by the autonomic nervous system, HR has been widely applied to assess physiological responses to environmental and management-related stressors. For instance, Marín et al. (2025) analysed HR as a physiological parameter associated with animal welfare in cattle, while Niccolai et al. (2024) investigated the relationship between HR and dynamic body acceleration in free-ranging cattle. Zapata Cardona et al. (2024) also considered HR in combination with hormonal indicators to assess stress responses in pigs. In parallel, several technological studies investigated wearable sensor systems for continuous HR monitoring (Pereira et al. 2019; Youssef et al. 2020), and review papers consistently recognise HR as a key physiological parameter for livestock welfare assessment (Neethirajan 2020; Neethirajan et al. 2021; Neethirajan and Kemp 2021). The retrieved literature indicates that continuous HR monitoring is increasingly feasible through wearable biosensors, enabling real-time assessment of cardiovascular responses without repeated manual measurements. This represents a major advantage for Precision Livestock Farming by facilitating continuous welfare surveillance under commercial conditions. However, practical implementation remains constrained by sensor attachment, limited battery life, signal degradation due to motion artefacts, and the need to maintain reliable skin or body contact. In addition, because HR is influenced by multiple physiological and behavioural factors, including physical activity, thermal load, fear, pain, and handling, it lacks specificity as a standalone physiological measure of animal welfare and should be interpreted alongside complementary physiological, behavioural, or environmental measures.

Closely related to cardiovascular activity, heart rate variability (HRV) was reported in 4 studies. Wierig et al. (2018) investigated the use of smartphone-based systems to record HRV in dairy cows, while Neethirajan et al. (2021) and Neethirajan and Kemp (2021) identified HRV as a promising physiological parameter in the context of sensor-based monitoring and digital phenotyping. Unlike HR, HRV quantifies beat-to-beat variations in heart rate and provides information on autonomic nervous system regulation through the balance between sympathetic and parasympathetic nervous activity; this offers the distinct advantage of greater biological specificity, providing a highly sensitive and nuanced understanding of emotional states, chronic stress, and adaptive capacity. However, its application in commercial PLF remains severely limited by the technical challenges of data acquisition; calculating accurate beat-to-beat intervals requires exceptionally clean, high-frequency electrocardiographic signals and advanced processing algorithms, making continuous HRV monitoring highly susceptible to disruption and data loss from even minor sensor displacement or motion artefacts.

Respiratory rate (RR) was reported in 20 studies and was primarily associated with thermal stress assessment. Because respiratory responses rapidly change under heat stress conditions, RR represents a sensitive physiological parameter of environmental challenges. Recent studies applied non-contact and computer vision-based approaches to monitor respiratory patterns in sheep and dairy cattle (Bonafini et al. 2024; Shu et al. 2024), while earlier research also identified RR as a key parameter for evaluating heat stress responses (Mostaço et al., 2015; de Sousa et al. 2016; de Oliveira et al. 2018). This relevance is further supported by review studies focusing on thermal physiology and livestock welfare (Sejian et al. 2021, 2022; Colditz 2022). Evidence from the included studies suggests that contact-free monitoring technologies have substantially improved the feasibility of continuous RR assessment within PLF by enabling objective, scalable, and non-invasive monitoring while reducing the subjectivity associated with manual respiratory counts. However, measurement accuracy remains dependent on image quality and can be compromised by animal movement, body positioning, occlusion, and environmental conditions, particularly under commercial farming conditions.

Glucocorticoids, including cortisol in mammals and corticosterone in birds, were widely reported as endocrine biomarkers of stress and welfare status. Different biological matrices, including saliva, hair, and faeces, have been used to assess both acute and chronic activation of the hypothalamic–pituitary–adrenal (HPA) axis across livestock species (Narayan et al. 2018; Wolf et al. 2020; Caulfield and Padula 2020; Hayes et al. 2021; Schütz et al. 2024; Abraham et al. 2024; Christophe et al. 2025). Several review studies further support the relevance of these endocrine biomarkers for welfare assessment and stress monitoring in livestock systems (Polsky and von Keyserlingk 2017; Galán et al. 2018; Sejian et al. 2021). Sampling these alternative matrices provides valuable insights into both short- and long-term physiological responses while avoiding the additional stress associated with blood collection. However, the routine integration of glucocorticoid measurements into PLF remains limited because laboratory analyses, sampling procedures, circadian variation, individual biological variability, and the delayed physiological response of glucocorticoids restrict their suitability for continuous, real-time welfare monitoring. In addition, hair and faecal analyses are better suited for retrospective assessment of long-term HPA axis activity than for supporting immediate management interventions.

Additional physiological biomarkers were less frequently investigated but highlighted emerging directions for non-invasive welfare assessment. For example, salivary oxytocin has been proposed as a biomarker associated with positive social interactions and emotional states (Moscovice et al. 2024), whereas salivary calprotectin has shown potential as an inflammatory marker associated with stress responses in pigs (López-Martínez et al. 2023). Other studies further explored complementary aspects of physiological monitoring, stress responses, and integrated welfare assessment approaches across different livestock systems (Doerfler et al. 2016; Seixas et al. 2017; Giergiel et al. 2021, 2022; Campbell et al. 2022; Leliveld et al. 2023; Shu et al. 2023; Li et al. 2024; Jardim et al. 2024). Although these emerging biomarkers show promise for broadening the physiological assessment of animal welfare, their application remains limited by the need for further biological validation, methodological standardisation, and technological development before they can be routinely incorporated into commercial PLF systems.

Overall, the physiological parameters identified in the reviewed literature differ considerably in both their biological specificity and technological maturity. While body surface temperature, heart rate, and respiratory rate have become increasingly compatible with continuous PLF monitoring through non-invasive sensing technologies, endocrine and emerging biomarkers remain more dependent on laboratory-based analyses and currently have limited applicability for real-time monitoring. These findings reinforce that no single physiological parameter can comprehensively assess animal welfare, highlighting the need for multimodal PLF systems that integrate complementary physiological parameters with behavioural, environmental, and management-related welfare indicators to support more robust and biologically meaningful welfare assessment.

### 3.3 Application of Precision Livestock Farming technologies within EU animal welfare indicator framework

To further explore the relationship between physiological parameters relevant to animal welfare and digital monitoring technologies, the studies retrieved through the second search string (‘*Animal Welfare AND Precision Livestock Farming*’) were analysed by mapping the monitored variables to the key animal welfare indicators proposed by the European Commission. These indicators include mortality and morbidity rates, antimicrobial use, lameness, injuries, abnormal behaviours, stocking density, and human-animal interactions, and are used to assess welfare outcomes at the farm level (European Commission 2024). According to the adopted assessment approach, these indicators can be classified into three main categories: animal-based indicators, measured directly on animals (e.g. behavioural changes, physical condition or health status); resource-based indicators, which describe environmental conditions such as available space or bedding; and management-based indicators, referring to husbandry practices applied on farms.

Among the 144 studies included in this systematic review, 100 were associated with the second search string (‘*Animal Welfare AND Precision Livestock Farming*’) and were therefore included in the analysis of PLF applications across the European Union animal welfare indicators. Table 5 summarises the distribution of PLF applications according to the corresponding European Union animal welfare indicators, the livestock species investigated, and the principal monitoring technologies employed.

**Table 5.** Distribution of Precision Livestock Farming (PLF) applications identified in the reviewed studies according to the European Union animal welfare indicators, livestock species, and principal monitoring technologies.

Welfare indicator category	EU welfare indicator	No. of PLF studies	Main species investigated	Main monitoring technologies
**Animal-based indicators**	Expression of social behaviour	27	Cattle, pigs, poultry, small ruminants	Computer vision, closed-circuit television camera (CCTV systems), acoustic monitoring
	Comfort around resting	3	Pigs, cattle, sheep	Accelerometers, posture sensors
	Absence of disease	13	Cattle, pigs, poultry	Computer vision, physiological sensors
	Locomotion score	12	Cattle, pigs	Accelerometers, pressure sensors, computer vision
	Body condition score (BCS)	8	Dairy cattle, goats, poultry	Computer vision, deep learning
	Metabolic health	3	Dairy cattle	Milk analysers, infra-red spectroscopy
	Good human–animal relationship	Not reported	–	–
	Mortality rate	5	Poultry, pigs	Audio monitoring, cameras
	Absence of injuries	5	Poultry, cattle	Camera-based detection systems
**Resource-based indicators**	Thermal stress	20	Cattle, pigs, poultry	Infra-red thermography, environmental sensors
	Indoor density	1	Pigs	Image analysis
**Management-based indicators**	Individual or group housing	3	Pigs, poultry, cattle	Cameras, behavioural monitoring
	Antimicrobial use	Not reported	–	–

Source: Author’s elaboration.

Notably, no studies included in this review explicitly addressed antimicrobial use or the human–animal relationship using PLF technologies. This finding suggests that current PLF research tends to emphasise welfare animal-based welfare indicators that are readily amenable to continuous, sensor-based monitoring, particularly behavioural measures, locomotion, thermal status, and physiological parameters. In contrast, antimicrobial use is generally documented through electronic farm management systems, veterinary treatment records, or prescription databases rather than through animal-centred sensing technologies, which may partly explain its limited representation within the sensor-oriented PLF literature identified in this review. Likewise, the human–animal relationship is a highly complex, multidimensional welfare construct encompassing behavioural, affective, and contextual components, for which standardised automated monitoring approaches remain limited. Consequently, management- and resource-based indicators were less frequently represented than animal-based indicators in the reviewed PLF applications.

Overall, the distribution of studies across welfare categories revealed notable differences in both research focus and technological development, suggesting opportunities for future PLF systems to better integrate sensor-derived information with farm management records and complementary data sources in support of more comprehensive animal welfare assessment. The following sections discuss the most frequently investigated welfare indicators and their associated PLF applications, with particular emphasis on the integration of physiological measurements into automated welfare monitoring systems.

#### 3.3.1 Expression of social behaviour

The expression of social and individual behaviours, including feeding, drinking, locomotion, resting, and social interactions, was the most frequently investigated animal-based welfare indicator in PLF research. This predominance reflects the increasing capacity of digital technologies to continuously and non-invasively quantify behavioural responses that provide indirect information on animal health, welfare, and adaptation to environmental conditions. Across the reviewed studies, computer vision, wearable sensors, acoustic monitoring, and machine learning algorithms were the principal technologies used to automatically detect and classify behavioural patterns. These technologies offer important advantages, including continuous, objective, and large-scale monitoring without disturbing the animals. However, although these approaches have substantially improved the objectivity and frequency of behavioural assessment, they primarily quantify observable behavioural responses rather than the biological processes underlying them. Behavioural changes are often non-specific and may result from multiple environmental, management, social, or health-related factors. Consequently, the routine integration of physiological measurements capable of validating whether behavioural alterations reflect underlying biological changes remains limited, highlighting an important opportunity for developing more comprehensive multimodal welfare monitoring systems.

Several studies have evaluated automated monitoring tools across a wide range of production systems and management scenarios. Automated technologies have been used to evaluate behavioural responses to virtual fencing (de Bruijn et al. 2025), which provides flexible grazing management without physical barriers but requires an adaptation period during which animals learn to associate auditory cues with virtual boundaries. Feeding behaviour has also been monitored during critical production stages such as weaning (Spina et al. 2024) and crop-livestock-forest integration systems (Moraes et al. 2024). At the group level, researchers have also examined factors shaping social interactions and behavioural patterns, such as kinship and hierarchical structures (Marina et al. 2024a, 2024b), while at the individual level, drinking behaviour and dominance relationships have been investigated using electronic drinkers and video-tracking systems, respectively (Nizzi et al. 2024). Radio Frequency Identification, RFID-enabled monitoring systems provide precise information on individual resource use and facilitate the assessment of social interactions; however, they require dedicated infrastructure and record animal behaviour only when individuals interact with instrumented stations (Nizzi et al. 2024).

Computer vision and machine learning approaches currently represent the dominant technological solutions for automated behavioural monitoring, primarily because they offer highly scalable, non-invasive observation that captures both the animal and its environmental context simultaneously. Deep learning models have been increasingly applied for the real-time detection and classification of different behavioural states in several livestock species (Fuentes et al. 2023; Lee et al. 2024; Saifudin et al. 2024; Ozella et al. 2024; Zhang et al. 2024), and their effectiveness has been confirmed by recent systematic evidence (Rohan et al. 2024). Their implementation in commercial farming environments has expanded considerably, as demonstrated by automated video surveillance systems (Kurras and Jakob 2024), while multi-view and spatiotemporal models have further improved detection accuracy and behavioural interpretation (Fuentes et al. 2023; Zhang et al. 2024). Nevertheless, the performance of vision-based systems may be affected by animal occlusion, stocking density, lighting conditions, dust accumulation, camera positioning, and the need for large annotated datasets to train robust machine learning models capable of generalising across different farming environments.

Wearable technologies, including accelerometers and inertial sensors, have complemented vision-based systems by enabling continuous tracking of activity patterns and movement dynamics (Balasso et al. 2023; Li and Chai 2023) at the individual animal level. Unlike camera-based systems, wearable sensors are less affected by environmental lighting conditions or visual occlusions and can provide detailed information on individual movement and behaviour. When integrated with positioning technologies, these sensors facilitate the analysis of movement trajectories, contact networks, and social interactions, providing additional insight into behavioural dynamics that are difficult to capture through image analysis alone (Benaissa et al. 2023; Moravcsíková et al. 2024; Walker et al. 2024). However, their practical implementation remains constrained by sensor attachment, battery life, maintenance requirements, and the risk of skin irritation, device loss or damage, and prohibitive costs for scaling across very large commercial herds. These systems have also been successfully applied to detect behavioural changes preceding reproductive events, including automated kidding detection in goats (Gonçalves et al. 2024), and calving prediction in dairy cattle through posture analysis (Mg et al. 2025).

Acoustic monitoring further complements behavioural assessment by enabling the detection of vocalisation patterns related to social interactions, distress, and stress-related responses (Van Erp-van der Kooij et al. 2023; Pann et al. 2024). As a completely non-contact technology, acoustic monitoring can support the early detection of behavioural and respiratory alterations. However, its performance may be influenced by background noise, housing acoustics, overlapping vocalisations, and difficulties in assigning vocalisations to individual animals within group-housed systems (Van Erp-van der Kooij et al. 2023; Pann et al. 2024).

The combination of complementary sensing technologies has facilitated the development of more robust automated behavioural monitoring systems for both research and commercial livestock production (Li and Chai 2023; Benaissa et al. 2023). Their reliability has also been validated against traditional observation methods, confirming their robustness under commercial farming conditions (Allueva Molina et al. 2023).

Behavioural monitoring has proven particularly valuable for assessing social hierarchy, competition, and welfare conditions. Some studies have analysed dominance relationships and resource access through the automatic detection of social interactions (Foris et al. 2019, 2020), while early warning systems have been developed to detect abnormal behaviours such as tail biting in pigs (D’Eath et al. 2018; Larsen et al. 2019). Furthermore, behavioural responses to innovative technologies, such as automated virtual fences, have been studied to assess adaptation processes in farming systems (Eftang et al. 2023).

#### 3.3.2 Thermal stress

Thermal stress represented another major research focus, with studies integrating environmental, physiological, and behavioural indicators.

Environmental monitoring systems provide continuous measurements of climatic variables, including ambient temperature, relative humidity, and heat load, enabling the real-time assessment of thermal conditions within livestock facilities (Flessner et al. 2024; Garcia et al. 2025). These systems provide continuous, objective environmental information and are relatively easy to implement at the farm level. However, environmental measurements alone do not reflect individual animal responses, as animals exposed to the same environmental conditions may exhibit different physiological adaptations depending on their species, production stage, health status, and acclimation capacity.

At the animal level, infra-red thermography (IRT) has become one of the principal technologies for evaluating physiological responses to heat stress through the measurement of body surface temperature and thermal patterns, while wearable sensors have complemented these observations by continuously recording behavioural and physiological responses (Aloia et al. 2024; Vercellino et al. 2025). Infra-red thermography offers important advantages because it is completely non-invasive, enables rapid screening of multiple animals, and can be readily integrated into automated imaging systems. Nevertheless, its accuracy is influenced by environmental conditions, including solar radiation, wind speed, ambient temperature, humidity, body region evaluated, hair coat characteristics, camera angle, and calibration procedures. On the other hand, as already mentioned, wearable sensors provide continuous individual monitoring but require sensor attachment, regular maintenance, and battery replacement, which may limit long-term implementation under commercial farming conditions (Aloia et al. 2024; Vercellino et al. 2025).

In parallel, machine learning algorithms have enabled the estimation of physiological parameters, including respiratory rate and body temperature, from image- and sensor-derived data (Wang et al. 2024; Li et al. 2024), together with behavioural indicators such as activity, posture, and aggregation patterns (Chopra et al. 2024; Massari et al. 2024). By integrating multiple data streams, machine learning improves the prediction of thermal stress and facilitates early detection of heat-related welfare impairment. However, model performance depends on the availability of large, high-quality datasets and may decrease when algorithms are applied to different species, breeds, housing systems, or climatic conditions without appropriate validation.

The reviewed studies consistently demonstrated that thermal stress responses vary substantially according to species, physiological status, production stage, and management conditions. This variability has been investigated across different production contexts, including transport conditions and commercial farming environments, highlighting the importance of context-specific monitoring approaches (Davison et al. 2020; Benni et al. 2020; Pacheco et al. 2020; Lovarelli et al. 2020; Ramón‐moragues et al. 2021; Schmeling et al. 2022; Piscopo et al. 2024; Machado et al. 2024; Bus et al. 2024; Alexy et al. 2024). Further contributions have demonstrated the value of integrated sensing platforms that combine environmental measurements with behavioural and physiological information to support more comprehensive welfare assessment and climate management (Leliveld et al. 2023; Ciliberti et al. 2025; Shafi et al. 2025). These multimodal approaches are highly advantageous because they mitigate the blind spots of single technologies by cross-validating environmental risk with actual biological responses, though they introduce significant data synchronisation and analytical complexities. Furthermore, advances in computer vision and data processing have enabled the non-invasive estimation of physiological responses associated with heat stress, including respiratory patterns and thermal dynamics, further strengthening the applicability of automated monitoring systems in livestock farming (Wang et al. 2024; Vercellino et al. 2025).

Overall, thermal stress monitoring represents one of the clearest examples of how integrating environmental information with physiological and behavioural measurements can improve welfare assessment. However, the substantial variability among species, production systems, and management conditions highlights the need for biologically validated and context-specific monitoring frameworks. These findings further support the integration of complementary physiological parameters with behavioural and environmental information to strengthen the interpretation of animal-based welfare indicators within PLF systems.

#### 3.3.3 Absence of disease

The absence of disease was increasingly addressed through early detection systems based on sensor data and predictive models. Specifically, machine learning has emerged as a key component of automated disease detection by integrating behavioural, physiological, and production data to identify patterns associated with impaired health (Džermeikaitė et al. 2023, 2025). By integrating heterogeneous data streams, machine learning facilitates continuous health surveillance and enables the early identification of subtle changes that may precede clinical disease before human observation. However, the predictive performance of these models depends on the quality, quantity, and representativeness of the training data and requires external validation before routine implementation across different livestock production systems. Additionally, real-time monitoring systems have enabled continuous health surveillance of individual animals (Michielon et al. 2024; Ouannes et al. 2025), facilitating earlier detection of health disturbances and supporting timely management interventions. These systems provide objective and continuous individual monitoring but require reliable sensor performance, data transmission, and infrastructure to ensure uninterrupted surveillance. Moreover, they can lack disease specificity, as various biological stressors can trigger similar behavioural changes (e.g. lethargy or reduced feed intake).

Most of the research has focused heavily on applying these digital tools to specific conditions, including respiratory diseases and udder health (Rosengart et al. 2022; Garrido et al. 2023), as well as the automated computer vision detection of lesions (Louton et al. 2022; Neethirajan 2022). Computer vision offers the advantage of non-invasive, automated assessment without handling animals, making it suitable for large-scale commercial applications. Nevertheless, lesion detection accuracy may be affected by lighting conditions, animal posture, body contamination, occlusion, and lesion visibility, while respiratory disease detection often relies on indirect behavioural or thermal proxies rather than direct physiological measurements.

Within these systems, behavioural and physiological measurements play a complementary role. Digital tracking of activity patterns, feeding behaviour, and social interactions serves as an indirect behavioural proxy for health status, whereas capturing underlying physiological responses provides direct biological evidence of homoeostatic disruption. Because both subtle behavioural alterations and physiological stress responses frequently precede the appearance of clinical signs, combining them significantly enhances predictive accuracy (Vázquez Diosdado et al. 2018; Fuchs et al. 2019; Ikurior et al. 2021). This multimodal approach reduces reliance on single indicators, which may lack specificity, and improves the biological interpretation of automated disease alerts.

Broader reviews further emphasise the role of digital technologies in disease prevention and herd health management, highlighting their potential to improve early diagnosis, support preventive interventions, and reduce production losses (Herlin et al. 2021; Shafi et al. 2025). However, despite substantial technological progress, integrating behavioural and physiological parameters remains essential to improve the biological interpretation of automated outputs and strengthen the reliability of disease detection systems for welfare assessment.

#### 3.3.4 Locomotion score

Locomotion score and related movement-based indicators are among the most extensively studied animal-based measures in PLF research, particularly for the detection of lameness, pain, discomfort, and impaired mobility. Because alterations in gait and movement often represent some of the earliest external manifestations of compromised health and welfare, continuous locomotion monitoring has become one of the most common applications of automated livestock surveillance.

Wearable sensors, accelerometers, inertial measurement units (IMUs), and activity trackers enable continuous quantification of movement patterns and activity levels, facilitating the early identification of locomotor alterations associated with health disorders (Pongsanun et al. 2025; Yao et al. 2025). These body-mounted devices offer the distinct advantage of providing continuous, highly precise 3D biomechanical data for individual animals regardless of their location, although they are limited by the physical stress of attachment, hardware loss, and battery life constraints.

In parallel, computer vision combined with deep learning has markedly improved the automated assessment of gait, posture, and locomotion, reducing the subjectivity of conventional visual scoring while increasing the scalability of lameness detection under commercial farming condition (de Paula et al. 2024; Kroese et al. 2025; Rajagukguk et al. 2025). Vision-based systems are highly advantageous as they are completely non-invasive and can assess multiple animals simultaneously, making them well suited for commercial applications; however, their primary limitation is the requirement for clear, unobstructed camera angles, which are often difficult to maintain in crowded pens, as well as a high sensitivity to environmental degradation such as poor lighting or dirty lenses.

Beyond lameness detection, movement-related digital data have been incorporated into broader welfare assessment systems to evaluate grazing behaviour, activity patterns, and behavioural changes associated with physiological status and reproductive events (Nyamuryekung’e et al. 2023; Mg et al. 2025). The integration of locomotion data with behavioural and physiological measurements further improves the interpretation of movement alterations and reduces the likelihood of false-positive welfare alerts arising from normal management or reproductive events.

These tracking technologies and artificial intelligence-based systems have been applied across multiple livestock species, including poultry, swine, and cattle, demonstrating the versatility of automated locomotion monitoring approaches (Hofstra et al. 2022; Olejnik et al. 2022; Siriani et al. 2023; Neethirajan 2023). While these multi-species AI tracking frameworks provide remarkable versatility in adapting to different farm environments, their accuracy is heavily dependent on robust digital infrastructure and substantial computing power to process continuous video or sensor streams.

Despite their potential, the large-scale implementation of locomotion monitoring systems remains constrained by economic factors, including equipment costs, infrastructure requirements, and technology adoption barriers at the farm level (Selvaggi et al. 2024). These limitations highlight the need for cost-effective and operationally feasible solutions capable of integrating movement-based information with physiological and behavioural parameters to improve welfare assessment.

#### 3.3.5 Body condition score (BCS)

Body condition score (BCS) is one of the most widely used animal-based welfare indicators because it reflects the animal’s nutritional status, metabolic balance, health condition, and long-term adaptation to environmental and management conditions (Linstädt et al. 2024). Variations in BCS may indicate compromised welfare associated with inadequate nutrition, disease, reproductive challenges, or chronic stress, making its assessment particularly relevant in livestock production systems.

Recent advances in computer vision, three-dimensional imaging, and deep learning have enabled the automated, non-invasive assessment of BCS, substantially reducing the subjectivity, labour requirements, and inter-observer variability associated with conventional visual scoring. While standard 2D imaging offers a cost-effective, easily deployable solution, 3D imaging provides the distinct advantage of capturing depth and volumetric data, making it highly resilient to variations in coat colour or pattern, though it requires more expensive hardware and complex spatial calibration.

Imaging-based models have demonstrated high accuracy for estimating BCS in dairy cattle (Yukun et al. 2019; Zheng et al. 2024; Lewis et al. 2025), while other investigations highlight their applicability across multiple livestock species (Temenos et al. 2024). These approaches are frequently integrated with behavioural monitoring and management systems to improve decision-making and animal monitoring capabilities (Silva et al. 2021; Giannone et al. 2025; Adli et al. 2025).

Automated BCS assessment offers considerable potential for continuous welfare monitoring because it enables non-invasive, objective, and scalable evaluation under commercial farming conditions without the stress of physical animal restraint. However, several limitations remain. The accuracy of image-based BCS estimation may be affected by lighting conditions, camera positioning, animal posture, and breed-specific body conformation, requiring robust calibration and species-specific algorithms to ensure reliable performance across different production systems (Silva et al. 2021). In addition, deep learning models require large, high-quality annotated datasets for training and validation, while their performance may decrease when applied to different farm environments or animals with extreme body condition scores. Furthermore, BCS primarily reflects cumulative nutritional and metabolic status rather than acute physiological responses, limiting its ability to detect rapid welfare changes. Consequently, BCS should be interpreted alongside complementary behavioural, physiological, and environmental indicators to provide a more comprehensive and biologically meaningful assessment of animal welfare (Schillings et al. 2023).

#### 3.3.6 Metabolic health

Metabolic health monitoring increasingly relies on indirect indicators generated from multiple data sources. Milk composition analysis combined with machine learning techniques has shown considerable potential for predicting blood metabolic profiles, enabling the early identification of metabolic alterations through routine, non-invasive measurements obtained during milking (Giannuzzi et al. 2022). This approach offers the significant operational advantage of integrating metabolic assessment into existing milking routines without requiring additional animal handling or stressful blood sampling. Similarly, sensor-derived activity data analysed using machine learning algorithms can identify behavioural patterns associated with physiological events, changes in health status, and disease progression, thereby supporting the early detection of metabolic imbalances (Lardy et al. 2023). The primary benefit of these wearable sensors is their ability to continuously capture subtle, systemic shifts in an animal's daily routine long before clinical symptoms manifest. Additional approaches, including thermal imaging and biometric sensing systems, enable continuous, contactless monitoring of physiological changes associated with metabolic status at the individual animal level (Mahato and Neethirajan 2025). These contactless systems are highly advantageous because they eliminate the wear-and-tear issues of physical sensors while providing immediate, objective physiological readouts.

Automated metabolic health assessment remains challenging because current PLF systems predominantly infer metabolic status from indirect proxy indicators rather than direct measurements of metabolic function. Giannuzzi et al. (2022) reported that the predictive performance of milk spectroscopy varies among individual metabolites, indicating that while some metabolic parameters can be estimated with high precision, others lack the accuracy required for reliable clinical diagnosis without blood validation. Likewise, behavioural parameters derived from activity sensors are not specific to metabolic disorders and may also reflect environmental conditions, reproductive status, management practices, or other health-related events (Lardy et al. 2023). Furthermore, the performance of thermal imaging and biometric sensing systems can be severely influenced by environmental conditions, sensor calibration, and data quality, while each technology captures only a limited aspect of the animal's physiological status (Mahato and Neethirajan 2025). Consequently, although these technologies provide valuable information for early metabolic surveillance, none independently offers a comprehensive assessment of metabolic function. These limitations highlight the need for multimodal PLF systems that integrate physiological, behavioural, and environmental information to improve the accuracy, biological validity, and practical applicability of automated metabolic health monitoring for animal welfare assessment.

#### 3.3.7 Absence of injuries

The assessment of injuries and physical damage increasingly relies on automated monitoring systems integrating visual, behavioural, and acoustic data sources. Computer vision models enable the detection and classification of physical injuries and external damage, particularly in poultry production systems (Jung et al. 2021; Bergamasco et al. 2025). These systems offer the advantages of continuous, non-invasive monitoring while reducing the subjectivity associated with manual welfare inspections. Their scalability also makes them suitable for commercial livestock production, where frequent individual animal assessment is often impractical.

Meanwhile, locomotion analysis supports the identification of gait alterations and lameness-related conditions (Kang et al. 2025), providing early, automated detection of biomechanical stress, even though it cannot independently diagnose the underlying physiological cause of the injury. In pigs, lesion detection has also been used as an early indicator of harmful social interactions, particularly tail-biting behaviour, enabling the identification of welfare issues before severe injuries occur (Larsen et al. 2019; Heseker et al. 2024). While highly effective for proactive intervention, the accuracy of these computer vision systems often degrades in densely packed pens where aggressive interactions and early-stage lesions are easily obscured from camera view.

Although automated lesion detection facilitates earlier intervention, its performance may be affected by lighting conditions, animal posture, body contamination, stocking density, and partial occlusion of injured body regions. Furthermore, because injuries often represent the outcome of previous health, behavioural, or environmental challenges, integrating lesion detection with physiological, behavioural, and environmental monitoring is essential to improve the biological interpretation of automated welfare assessments.

#### 3.3.8 Mortality rate

Mortality is an important animal-based welfare indicator that is primarily addressed through early-warning systems designed to identify distress signals, behavioural alterations, and critical events associated with health deterioration before death or culling occurs. By integrating multimodal data streams, including behavioural monitoring, acoustic surveillance, physiological parameters, and other sensor-derived information, these systems shift herd management from reactive mortality recording to proactive intervention, enabling the prediction of increased mortality risk and facilitating timely management actions (Mao et al. 2022; Sadeghi et al. 2023; Syafaah et al. 2024; Dawkins et al. 2025; Ferreira et al. 2025). The principal advantage of these systems is their ability to support early intervention through the continuous monitoring of behavioural and physiological changes associated with health deterioration. However, prediction models may generate false-positive alerts, increasing the risk of alarm fatigue among farm personnel and unnecessary interventions. Their performance also depends on the availability of reliable, continuous sensor data, robust digital infrastructure, and appropriate validation across different production systems and management conditions.

#### 3.3.9 Comfort around resting

Comfort around resting is comparatively less investigated than other welfare domains within PLF research. Current approaches primarily assess resting comfort through the monitoring of resting-related behaviours using sensors and computer vision systems, enabling the quantification of lying behaviour, posture, activity patterns, and space use (Walton et al. 2018; Pesenti Rossi et al. 2024; Liu et al., 2025). These systems offer the advantage of continuously and non-intrusively quantifying lying bouts and postural transitions, which are critical early indicators of both comfort and structural issues. However, computer vision often struggles with visual occlusions when animals huddle together to rest, and sensors alone frequently fail to differentiate between normal resting behaviour and pathological lethargy without additional physiological context. Consequently, they primarily quantify behavioural responses and do not directly assess the physical characteristics of the resting environment or the animal's physiological comfort during rest. Additionally, stocking density has been evaluated using computer vision approaches for animal counting and spatial distribution analysis, providing indirect information on space availability and resting opportunities (Feng et al. 2023). The primary benefit of this spatial tracking is its ability to map real-time space utilisation, revealing whether animals are crowding or avoiding specific microclimates within a pen. Conversely, they cannot independently determine whether resting conditions are comfortable, as housing design, bedding quality, flooring characteristics, and microclimatic conditions also influence resting behaviour and welfare.

Some studies have investigated housing conditions through tracking technologies and behavioural monitoring systems applied in both group- and individual-housing settings, aiming to evaluate how housing design and management practices influence animal behaviour and welfare outcomes (Wutke et al. 2021; Mehdizadeh et al. 2024; Gavojdian et al. 2024). These technologies provide objective, continuous assessments of animal–environment interactions, offering valuable insights into how housing design influences resource use, resting behaviour, and overall welfare. However, their implementation often requires dedicated monitoring infrastructure, and their performance and applicability may vary according to housing design, management practices, and production systems, limiting the direct transferability of models across different commercial settings.

## 4. Discussion

Precision Livestock Farming undoubtedly represents a major milestone in the evolution of the livestock production system, reflecting the transition toward data-driven approaches supported by sensing technologies, the Internet of Things (IoT), artificial intelligence (AI), and Big Data. This technological transformation is progressively reshaping both livestock housing systems and farm management strategies through the implementation of AI-driven and data-centric architectures (Silvestri et al. 2026). Although the concept of PLF was first introduced in the early 2000s during the First European Conference on Precision Agriculture, its development has accelerated substantially over the last two decades. Initial applications, including transponders for animal identification, robotic milking systems, and automated feeding technologies, have progressively evolved toward increasingly automated and interconnected farming systems supported by artificial intelligence and machine learning approaches. In this context, the adoption of sensor-based technologies for monitoring animal behaviour, physiology, production parameters, and environmental conditions has expanded alongside the increase in herd size and production intensification observed in modern livestock systems (Marino et al. 2023). More specifically, PLF systems have progressively evolved from simple identification and automated recording technologies, such as Radio-Frequency Identification systems, toward integrated digital monitoring architectures capable of generating continuous ‘digital representations’ of individual animals through the integration of physiological, behavioural, and environmental data (Norton et al. 2019). This transition from isolated measurements to multidimensional monitoring systems represents a major shift in PLF development, enabling more comprehensive animal assessment and increasingly individualised management strategies. Within this technological evolution, Edge AI is emerging as a promising advancement for PLF applications (Jiang et al. 2023). By processing data directly on devices such as accelerometers, cameras, and microphones, Edge AI reduces reliance on remote servers and cloud-based infrastructures, minimising data transmission requirements and latency. This decentralised approach facilitates near real-time analysis and faster decision-making, which is particularly relevant for livestock systems where large volumes of continuously generated data require rapid processing to support timely interventions and the early detection of health and welfare issues.

The principal finding of this review is that the successful integration of physiological indicators into PLF depends not only on their biological relevance but also on their operational feasibility for continuous, automated monitoring. Although physiological measurements provide direct information on homoeostatic regulation and stress responses (Polsky and von Keyserlingk 2017; Colditz 2022), substantial differences exist in their compatibility with current sensing technologies. Body surface temperature, heart rate, and respiratory rate are increasingly compatible with non-invasive monitoring through infra-red thermography, wearable biosensors, and computer vision (Bonafini et al. 2024; Song et al. 2025), whereas heart rate variability and endocrine biomarkers remain constrained by signal quality requirements, laboratory analyses, and methodological complexity (Neethirajan et al. 2021; Neethirajan and Kemp 2021), (Table 6).

**Table 6.** Comparative overview of physiological indicators for PLF-based animal welfare assessment.

Physiological indicator	Biological relevance for welfare assessment	Measurement approach	Main advantages	Main limitations
**Body surface temperature (infra-red thermography - IRT)**	Reflects thermoregulation, health status, inflammation, and thermal stress responses	Infra-red cameras, thermal sensors	Non-invasive, rapid, suitable for automated monitoring and AI-based analysis	Influenced by environmental conditions, distance, anatomical region, and calibration
**Respiratory rate (RR)**	Sensitive indicator of thermal stress and respiratory disorders	Computer vision, thermal imaging, acoustic monitoring, accelerometer-based collars	Non-invasive, rapid physiological response; possible non-contact measurement	Often measured through visual observation, requires accurate detection of respiratory movements; affected by posture and image quality
**Heart rate (HR)**	Cardiovascular responses to stress, behaviour, activity, traits related to metabolism	Wearable sensors, ECG-based devices, portable monitors	Non-invasive, direct physiological information; useful for acute stress assessment and welfare monitoring	Transmission between the logger and the sensor by infra-red, sensor attachment, battery life constraints, animal tolerance, signal artefacts, individual monitoring requirements
**Heart rate variability (HRV)**	Cardiovascular responses, provides information on autonomic nervous system	ECG or wearable cardiac sensors, portable systems	Non-invasive, more informative than HR alone for stress, emotional states, temperament and resilience	Transmission between the logger and the sensor by infra-red, sensor attachment, battery life constraints, animal tolerance, signal artefacts, individual monitoring requirements signals and advanced processing
**Glucocorticoids (cortisol/corticosterone)**	Endocrine biomarkers, established indicators of endocrine stress response and welfare status	Biological sampling (saliva, faeces, hair and blood)	Non-invasive, strong biological validation; suitable for acute and chronic stress assessment	Sampling procedures, laboratory analysis, limited real-time capability
**Oxytocin**	Potential indicator of positive social interactions and emotional states	Biological sampling (saliva, blood and milk)	Non-invasive, provides information beyond negative stress responses	Sampling procedures, laboratory analysis, limited real-time capability, limited validation
**Calprotectin (Inflammatory biomarkers)**	Inflammatory marker, potential indicators of physiological monitoring and stress	Biological sampling (saliva and blood)	Non-invasive, potential to assess the effect of different husbandry conditions and also to evaluate the homoeostasis	Sampling procedures, specific assay, clinical validation, limited field applicability and automation
**Integrated physiological profiles**	Combines multiple dimensions of animal state	Multimodal sensing and AI fusion	Enables holistic welfare assessment and predictive analytics	Requires standardised protocols, data integration, and validation

These findings support previous observations that no single physiological indicator is sufficient to comprehensively assess animal welfare. Infra-red thermography enables rapid, contact-free thermal assessment but remains sensitive to environmental conditions. Heart rate can be continuously monitored using wearable biosensors but is influenced by multiple physiological and behavioural processes, reducing its specificity. Conversely, heart rate variability provides more specific information on autonomic regulation but remains technically demanding under commercial farming conditions. Similarly, glucocorticoids remain among the most biologically informative indicators of stress, although their dependence on laboratory analysis limits their integration into real-time PLF applications.

A key finding of this review is the strong predominance of animal-based indicators within PLF applications. In particular, behavioural monitoring represents the most advanced and widely studied area of research, reflecting the ability of computer vision, wearable sensors, and acoustic systems to capture complex animal responses across multiple contexts (Neethirajan 2023; Rohan et al. 2024). Supported by deep learning algorithms, these multisensor approaches have facilitated the automated detection of both individual behaviours, such as drinking (Ruchay et al. 2026), and social interactions (Fuentes et al. 2023; Benaissa et al. 2023; Lee et al. 2024). Recent computer vision applications further illustrate this potential, with AI-based systems enabling individual cattle identification and tracking across different farm environments (Mon et al. 2024), as well as automated posture analysis to identify behavioural patterns associated with normal and abnormal calving and support timely intervention (Khin et al. 2024). Similarly, thermal stress evaluation has emerged as one of the most developed application areas within PLF, as it successfully integrates environmental, physiological, and behavioural data streams to accurately characterise animal responses (Wang et al. 2024; Garcia et al. 2025; Vercellino et al. 2025). The findings of this review reinforce the growing consensus that multimodal monitoring provides a more biologically meaningful assessment of welfare than single-indicator approaches. Linstädt et al. (2024) emphasised the importance of combining biologically valid and operationally feasible indicators for practical welfare assessment, whereas Axiu et al. (2026) demonstrated that integrating thermal imaging, movement data, acoustic signals, and physiological measurements improves disease detection, reproductive management, and environmental control.

The increasing adoption of IoT-based infrastructures, predictive analytics, and artificial intelligence suggests a progressive transition towards integrated monitoring architectures capable of supporting proactive welfare management (Li and Chai 2023; Michielon et al. 2024; Shafi et al. 2025). Nevertheless, widespread implementation will require greater interoperability among monitoring platforms, standardised data formats, and rigorous biological validation of automated indicators before routine application in commercial livestock systems (Allueva et al. 2023; Neethirajan and Kemp 2021).

Despite these technological advances, the present review revealed a significant imbalance among welfare indicator categories. Resource and management-based indicators were considerably less represented than animal-based indicators within the reviewed PLF applications. Notably, no retrieved studies explicitly addressed antimicrobial use or the human–animal relationship through PLF technologies, despite their inclusion in contemporary European animal welfare assessment frameworks (European Commission 2024). Their limited representation likely reflects both technological constraints and the difficulty of converting complex management practices and human-related factors into objective, continuously measurable variables. As a consequence, the current predominance of animal-based monitoring risks providing only a partial representation of welfare status at the farm level. A truly comprehensive PLF-based welfare assessment will require the integration of animal, resource, and management-based indicators within unified assessment frameworks capable of capturing the multidimensional nature of animal welfare.

From an applied perspective, PLF technologies offer considerable potential to support objective, continuous, and non-invasive welfare assessment under commercial farming conditions. However, their successful implementation will depend not only on technological advances but also on the development of standardised methodologies, robust biological validation, and greater interoperability between sensing platforms and farm management systems. Future research should therefore prioritise the development of multimodal monitoring frameworks capable of integrating physiological, behavioural, environmental, and management-related information into biologically meaningful and actionable decision-support tools. Further validation across livestock species, production systems, and farming contexts will also be necessary to improve the robustness, transferability, and practical applicability of these technologies.

The findings of this review should be interpreted in light of certain methodological considerations. First, the literature search was restricted to a single scientific database. Furthermore, given the considerable heterogeneity in livestock species, study designs, physiological indicators, and PLF technologies, a formal assessment of study quality or risk of bias was not conducted. Consequently, the present findings should be regarded as a qualitative synthesis of current evidence and research trends rather than a quantitative synthesis of the available literature.

Finally, recent scientometric evidence suggests that social science dimensions are becoming increasingly relevant within PLF research (Jiang et al. 2023). Beyond technical performance, the growing digitalisation of livestock systems also raises important ethical and societal considerations. Although automated monitoring technologies substantially improve the objectivity and continuity of welfare assessment, important aspects of animal welfare, including affective states, social interactions, and stockperson–animal relationships, remain difficult to quantify using sensor technologies alone. In this context, the principal value of PLF lies in its ability to function as a decision-support tool that complements, rather than replaces, veterinary expertise, husbandry practices, and positive human–animal interactions within comprehensive animal welfare assessment frameworks.

## 5. Conclusions

Within European context, the 13 welfare indicators examined in this review represent key components of contemporary animal welfare assessment frameworks. While these indicators have traditionally been evaluated through standardised on-farm protocols conducted by trained personnel, many are now being incorporated into PLF systems, enabling more objective, continuous, and real-time monitoring of animal health and welfare.

The findings of this review demonstrate that physiological measurements provide valuable biological information that complements behavioural, production-related, and environmental measures within PLF-based welfare assessment. Among the physiological parameters reviewed, body surface temperature, heart rate, and respiratory rate currently appear to be the most operationally feasible for routine implementation, particularly through non-invasive technologies such as infra-red thermography, wearable biosensors, and computer vision. Nevertheless, no single physiological parameter is sufficient to comprehensively characterise animal welfare, highlighting the importance of integrating complementary sources of information.

Overall, this review indicates that the future contribution of PLF to animal welfare assessment will depend on the integration of physiological, behavioural, environmental, and management-related data within multimodal monitoring frameworks capable of supporting scientifically robust and practically applicable welfare assessment. Such an integrated approach is consistent with the policy direction recently outlined by the European Commission for the future of EU agriculture and livestock production. The Vision for Agriculture and Food identifies research, innovation, digitalisation, and knowledge exchange as essential components of a competitive, resilient, and future-oriented agricultural sector, while also reaffirming the need to maintain and further develop high standards of animal health and welfare (European Commission 2025a). These priorities are reflected in the proposed framework for the CAP 2028-2034, which is expected to continue supporting investments, innovation, advisory services, and technological development as enabling conditions for the transition towards more sustainable and resilient farming systems (European Commission 2025b). More specifically, the recently adopted EU Livestock Strategy recognises animal health and welfare as integral components of the long-term sustainability and resilience of European livestock production and highlights the potential contribution of Precision Livestock Farming, data-driven management, and digital early-warning systems to the development of more efficient and responsive production systems (European Commission 2026a). Its accompanying Staff Working Document further identifies Precision Livestock Farming and data-based decision-making among the technological developments capable of supporting improved farm management, disease prevention, resource-use efficiency, and animal care, while also acknowledging the need to facilitate their uptake across different livestock production systems (European Commission 2026b).

In this policy context, the findings of the present review suggest that future updates to EU animal welfare frameworks, including those developed within the CAP, should move beyond the adoption of individual sensing technologies and promote multimodal monitoring frameworks capable of integrating physiological and behavioural data with environmental and management-related information. Multimodal data integration (e.g. combining environmental IoT sensors with computer vision) could facilitate the earlier identification of adverse health and welfare conditions, improve the interpretation of physiological responses within their behavioural and environmental context, and provide more continuous and objective evidence to complement periodic on-farm assessments. However, the policy relevance of these systems will depend not only on their technical performance but also on the development of scientifically validated indicators, harmonised assessment criteria, transparent and interpretable algorithms, and appropriate data-governance arrangements. Addressing these requirements would strengthen the reliability, practical applicability, and regulatory relevance of PLF-based welfare assessment and support its future integration into European animal welfare monitoring and policy frameworks.

## Data Availability

The authors confirm that the data supporting the findings of this study are available within the article.
